# The effect of non-adherence to antipsychotic treatment on rehospitalization in patients with psychotic disorders

**DOI:** 10.1177/20451253211027449

**Published:** 2021-06-25

**Authors:** H. Abdullah-Koolmees, S. Nawzad, T.C.G. Egberts, J. Vuyk, H. Gardarsdottir, E.R. Heerdink

**Affiliations:** Department of Pharmacoepidemiology and Clinical Pharmacology, Utrecht Institute for Pharmaceutical Sciences, Faculty of Science, Utrecht University, Postbus 85500, Utrecht 3508 GA, The Netherlands; Department of Clinical Pharmacy, University Medical Center Utrecht, Utrecht, The Netherlands; Department of Clinical Pharmacy, University Medical Center Utrecht, Utrecht, The Netherlands; Department of Pharmacoepidemiology and Clinical Pharmacology, Utrecht Institute for Pharmaceutical Sciences, Faculty of Science, Utrecht University, Utrecht, The Netherlands; Division of Willem Arntsz, Altrecht Institute for Mental Health Care, Utrecht, The Netherlands; Department of Pharmacoepidemiology and Clinical Pharmacology, Utrecht Institute for Pharmaceutical Sciences, Faculty of Science, Utrecht University, Utrecht, The Netherlands; Department of Pharmacoepidemiology and Clinical Pharmacology, Utrecht Institute for Pharmaceutical Sciences, Faculty of Science, Utrecht University, Utrecht, The Netherlands

**Keywords:** adherence, antipsychotic medication, continued drug use (implementation), discontinuation, initiation, non-adherence, patients with psychotic disorders, phases of medication use, rehospitalization

## Abstract

**Background and Aims:**

Many patients with psychotic disorders are non-adherent to antipsychotic (AP) medication(s), potentially contributing to rehospitalization. It is unknown whether non-adherence in different phases of AP use is associated with rehospitalization. The aim of this study was to assess the association between non-adherence to APs and rehospitalization in patients with psychotic disorders. Non-adherence was assessed specifically for the initiation, continued drug use and early discontinuation of AP use.

**Methods:**

A retrospective follow-up study was performed. Adult patients were included at discharge if they suffered from schizophrenia, psychotic, or bipolar I disorder; had been hospitalized in a psychiatric hospital for ⩾7 days; and were treated with oral APs. Patients discharged between January 2006 and December 2009 from Altrecht Mental Health Care were included. Non-adherence was studied in the three phases of medication use: initiation, continued drug use (implementation) and (early) discontinuation after discharge until the end of follow up or until patients were rehospitalized. Cox regression analysis was used to assess the strength of the association between non-adherence for the different phases of AP use and rehospitalization during follow up and expressed as relative risk (RR) with 95% confidence intervals (CI).

**Results:**

A total of 417 patients were included. Patients who did not initiate their APs compared with those who did in the first month (RR = 1.62, 95% CI: 1.19–2.19) and between the first and third month after discharge (RR = 1.70, 95% CI: 1.04–2.79) had the highest risk for rehospitalization during follow up. Overall, patients who did not initiate their AP medication within the first year after discharge had a RR of 2.70 (95% CI: 1.97–3.68) for rehospitalization during follow up compared with those that initiated their AP.

**Conclusion:**

Not initiating APs right after discharge was associated with an increased risk of rehospitalization. Interventions should aim to promote the initiation of APs soon after discharge to minimize the risk of rehospitalization.

## Introduction

There is ample evidence for the effectiveness of antipsychotics (APs) in the treatment of schizophrenia in reducing the number of recurrent psychotic episodes.^1^ Adherence to APs is important; however, studies show that adherence is a problem in patients suffering from schizophrenia by indicating that about half of the patients are non-adherent to taking their AP medication.^2–4^ Reasons for non-adherence include, amongst others, lack of knowledge of the disease and/or the disease severity, the (fear of) side-effects, difficulty recognizing symptoms, non-effectiveness, not acknowledging the need for antispychotic therapy, or distrust in the effectiveness.^5–8^

Patients with treatment failure have a risk of relapse resulting in acute psychosis, leading to psychiatric (re)hospitalizations and considerable economic costs.^9–19^ Although Taylor and Jauhar reported that long-term outcomes including relapse and recovery in schizophrenia and psychosis have improved, relapse risk remains high.^20^ About half of the patients with schizophrenia have a relapse within a period of 2 years after their first psychotic episode.^13^ Earlier work from the authors showed that approximately one-third of patients with schizophrenia discharged from a psychiatric hospital and who were treated with APs were readmitted within a 6-month period. In addition, several studies showed that patients who are less adherent to AP therapy have at least twice the risk of psychiatric rehospitalization than those who adhere to AP therapy.^1,3,10,19,21^

Medication use is a dynamic process that can be divided into three phases: initiation, continued drug use/implementation (i.e., the patient’s rhythm of drug taking relative to the prescribed drug regimen), and discontinuation.^22^ The first phase, initiation, consists of starting (or not starting) to use the medication prescribed by the physician (e.g., psychiatrist). The second phase, continued drug use/implementation, describes the extent to which a patient’s actual dosing corresponds to the prescribed dosing regimen. In patients who initiate therapy, implementation can be observed/measured as continuous use or with gaps between AP prescriptions. The last phase, discontinuation, concerns (early) stopping of the medication without agreement with the psychiatrist.

Most research on adherence to AP medication adopt a more general approach for assessing adherence, for example, identifying patients being adherent or non-adherent during a predefined study period.^11–19,23,24^ By investigating adherence in a more detailed way, that is, by investigating adherence during each phase of medication treatment and investigating the association between medication use during these phases and psychiatric rehospitalization, interventions can be better tailored to target non-adherence and subsequently prevent psychiatric rehospitalizations.

The aim of this study was to assess the association between use of APs assessed during the three phases of medication use (i.e., initiation, continued drug use and discontinuation) and psychiatric rehospitalization after discharge.

## Methods

### Setting

The Psychiatric Case Register Middle Netherlands (PCR-MN) registers all in- and outpatient psychiatric care provided in the province of Utrecht, The Netherlands, including the Altrecht Mental Health Care (Altrecht).^1^ Altrecht is a conglomeration of four hospitals in the province of Utrecht in the middle region of The Netherlands. Medication is provided to inpatients by the Altrecht’s hospital pharmacy. The hospital files contain information data on unique patient number, gender, birth date, psychiatric diagnosis according to the Diagnostic and Statistical Manual of Mental Disorders-IV (DSM-IV), date of diagnosis, type of care (inpatient and outpatient), department of admission, start and end of admission, and medication use from 2006. For each patient, data on medication included the start and end date of use, type of medication used, and the prescribed dose. Information on outpatient medication use was available from the Achmea Health Database (Achmea). Achmea is the largest health insurance company in the middle region in The Netherlands, which includes the province of Utrecht. Therefore, only patients insured by Achmea health insurance were included for whom information on outpatient medication was available.^1^ Inpatient data was linked anonymously to outpatient data. Outpatient medication histories contained all outpatient dispensing information covering prescriptions from general practitioners and all other physicians. The outpatient medication history contained information about the date of dispensing and the nature and quantity of medication dispensed. Medication was coded according to the World Health Organization (WHO) anatomical therapeutic chemical (ATC) coding system.^11^ The study was approved by the institution’s scientific review board (11-61/oz1111/ck), and performed in accordance with The Federation of Dutch Medical Scientific Societies’ Code of Conduct for the use of data in Health Research.

### Design and study population

This retrospective follow-up study included all adult patients (⩾18 years) with a diagnosis of psychotic disorder (DSM-IV diagnosis codes 293, 295, 297.1, 297.3, 298.8, or 298.9) or bipolar I disorder (DSM-IV diagnosis code 296 excluding 296.89 and 296.9) who were discharged from Altrecht Mental Health Care between 1 January 2006 and 31 December 2009 and treated with AP medication during the week prior to discharge (ATC: N05A excluding lithium). For each patient, only the first psychiatric hospitalization of 7 days or longer during the study period was included.^19^ Psychiatric hospitalizations that occurred within 7 days following discharge where considered as belonging to the same psychiatric hospitalization.^16,18,19^ The study period included psychiatric hospitalization until psychiatric rehospitalization or the end of data collection whichever came first (=end of follow up).

### Outcome

The primary outcome of this study was the first psychiatric rehospitalization for any reason during follow up after discharge.^19^

### Determinant: non-adherence

Non-adherence was studied in the three phases of medication use: initiation, continued drug use (implementation), and early discontinuation.

#### Initiation

A patient was considered non-adherent in the initiation phase if the patient did not initiate the AP after discharge. In The Netherlands, AP medication for use after hospital discharge is dispensed by the community pharmacy. When patients are discharged just before or during the weekend, they might get AP medication covering a maximum of 2–3 days from the hospital. Therefore, patients are expected to fill prescription(s) at their community pharmacy during the first week after discharge. Initiation was considered when patients filled their AP prescription(s) after discharge at their community pharmacy. Initiation was assessed during the first week (reference), between the first week and first month, and between the first and third month following discharge. Initiation was defined as initiated or not initiated based on the date that the AP medication was dispensed from the community pharmacy.

#### Continued drug use

To assess AP use categories, the time after discharge was divided in episodes of 3 months as 3, 6, 9, and 12 months after discharge.

Continued drug use was assessed by measuring the medication possession ratio (MPR) for each patient (*N* of days of medication supplied within the refill interval/number of days in refill interval, %) for each episode at 3, 6, and 9 after discharge. Patients’ adherence was classified as high or low taking compliance. If the patients’ MPR was ⩾90%, the patients’ compliance was considered high and if MPR was <90%, the patient’ compliance was considered low. The theoretical duration of each dispensed AP prescription was estimated in days based on the number of units dispensed and the prescribed daily dose. Patients could get their AP medication dispensed too early or too late resulting in a gap or overlap of medication on hand. If a subsequent AP dispensing was dispensed prior to the theoretical end date of the previous dispensing, the number of overlapping days was added to the theoretical end date of the subsequent AP medication dispensing.^17^

#### Early discontinuation

Patients continuing (e.g., continuers) or early discontinuing (e.g., early discontinuers) AP medication were categorized as follows. Patients that refilled their AP medication with a gap of <7 days, were considered as continuers. If patients did not refill their AP medication again and had a gap of ⩾7 days, they were considered as early discontinuers and thus non-adherent.

#### AP use categories

Patient who initiated therapy were categorized on basis of their AP use into four categories as shown in Table 1. Patients who initiated their AP medication within 3 months after discharge were categorized in these groups as follows: continuers with a high taking compliance (reference), continuers with low taking compliance, discontinuers with high taking compliance, and discontinuers with low taking compliance. This was assessed for first 3 months after discharge. Additionally, this was also assessed for 6, 9, and 12 months after discharge. The risk of rehospitalization for the different categories was assessed during different time periods within the follow-up period, as described in the data analysis section.

**Table 1.** Patient classifications based on the continued drug use (implementation) and discontinuation phases of medication use.

	High taking compliance (MPR ⩾ 90%)	Low taking compliance (MPR < 90%)
Continuers (refill gap < 7 days)	Continuers with a high taking compliance (reference)	Continuers with low taking compliance
Discontinuers (refill gap ⩾7 days)	Discontinuers with high taking compliance	Discontinuers with low taking compliance

MPR, medication possession ratio.

#### Potential confounders

Stratification was performed for variables considered as potential confounders: gender, age at discharge, duration of hospitalization, dosage form of AP medication [only oral AP medication and depot (long-acting injectable AP) and oral AP medication], diagnoses (patients with schizophrenia *versus* without schizophrenia, and patients with a psychiatric disorder other than schizophrenia and bipolar disorder *versus* other patients). In addition, an extra stratification was performed by classifying the previously mentioned diagnosis groups by gender.^13–16^

#### Data analysis

**Figure 1.**
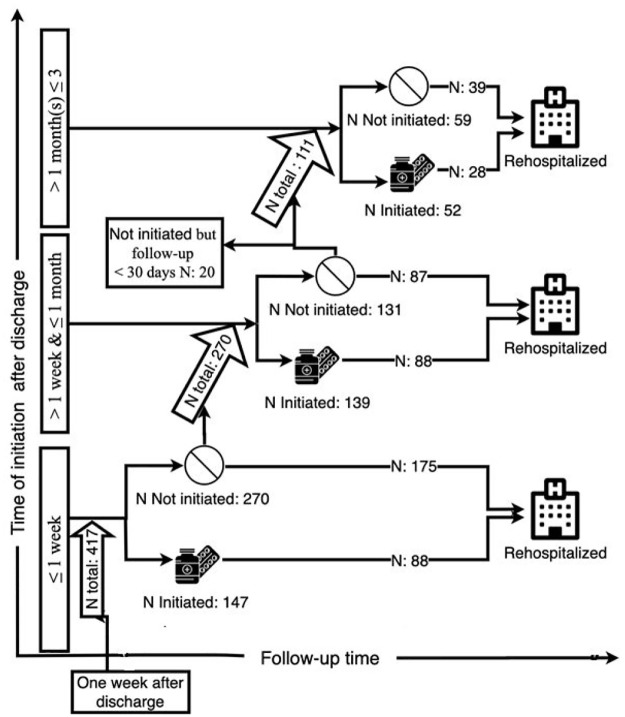
Number of patients who initiated (or not) their antipsychotic medication during different time periods for the first 3 months of follow up, and the portion of these patients who were rehospitalized at some point during the entire follow-up period in order to assess the association between initiation and rehospitalization.

The number of patients initiated AP medication was assessed at first week (reference), between the first week and first month, and between the first and third month following discharge. The number of patients who continued drug use (implementation), and discontinued AP medication was assessed for the first 3 months after discharge. Furthermore, these analysis were also performed for the episodes 6, 9, and 12 months after discharge as shown in Figure 1.

Cox regression was conducted to assess relative risk (RR) with time to rehospitalization or time till end of follow up. RR was assessed in order to investigate the association risk between non-adherence for the three phases of AP medication use and rehospitalization during follow up.

The RR of rehospitalization during follow up was determined for patients who did not initiate their AP medication within the first week, between the first week and first month, and between the first and third month following discharge compared with patients who did initiate their AP medication within the first week after discharge (reference). Furthermore, the risk was assessed within the first month by comparing patients that initiated AP medication between the first week and first month after discharge, and patients who did not initiate their AP medication during the first month to patients who initiated their AP medication within the first week (reference). In addition, the RR of rehospitalization for the patients in the different AP use categories (as shown in Table 1) was assessed for the continued drug use (implementation) and early discontinuations during the first 3 months, between the 3rd and 6th month, between the 6th and 9th month, and ultimately between the 9th and 12th month following discharge. Patients who were classified as continuers with a high taking compliance (MPR ⩾90%) were set as reference.

Data analysis was performed using IBM Software package SPSS (version 25.0).

## Results

A total of 417 patients was included. The mean age was 43.3 years [standard deviation (SD): 15.1], 265 (63.4%) were male, and most patients (70.5%) were diagnosed with schizophrenia, see Table 2. The median duration of psychiatric hospitalization was 66 days (range: 7–1410). All patients used oral AP medication, olanzapine and risperidone being mostly used, and 94 patients (22.5%) used both oral APs and depot long-acting injectable APs at discharge. Of the 417 patients, 376 (90.2%) refilled their AP medication after discharge. About two-thirds of the patients (63.1%) were rehospitalized during the study period.

**Table 2.** Patient characteristics at discharge.

Characteristics		*N*	%
Included patients	Total	417	100
Gender	Male	265	63.5
	Female	152	36.5
Age (years)	Mean ± SD	43.3 ± 15.1	
	Range	19–89	
	Median	41	
	19–32		25.9
	33–41		27.3
	42–53		23.3
	>53		23.5
Duration baseline hospitalization (days)	Mean ± SD	146 ± 10.7	
	Median	66	
	Range	7–1410	
Diagnosis of psychotic disorders	Schizophrenia (DSM-IV 295)	294	70.5
	Bipolar disorder (DSM-IV 296)	5	1.2
	Other: psychotic disorder due to medical condition, delusional disorder and brief psychotic disorder (DSM-IV 293, 297, 298)	118	28.3
Duration of follow up (days)	Mean ± SD	1089 ± 20	
	Range	365–1792	
Dosage form of antipsychotic	Oral antipsychotics	417	
	Depot long-acting injectable antipsychotic and oral antipsychotics	94	22.5
Patients rehospitalized	Total	263	63.1

SD, standard deviation.

### Initiation phase

Figure 1 and Tables 3 and 4 show the initiation status of AP medication within the first 1 and 3 months of follow up and rehospitalization; these numbers were used to assess the association between initiation and rehospitalization. Of the 417 patients, 35.3% (147) initiated their AP medication within the first week, 33.3% (139) eventually initiated use of APs between the first week and first month after discharge, and 31.4% (131) did initiate any AP medication after discharge. Of the 64.7% (270 of 417) who did not initiate their AP medication in the first week following discharge, 37.2% (175 of 471) were rehospitalized at some point during follow up.

**Table 3.** RRs for the association between the initiation phase of antipsychotic use and rehospitalization within the first month of follow up.

Time	*N* total (%)	*N* rehospitalized	*N* not rehospitalized	RR crude (95% CI)
Overall	417 (100)			
Initiated ⩽ 1 week	147 (35.3)	88	59	Reference
Initiated > 1 week and ⩽1 month	139 (33.3)	88	51	0.87 (0.64–1.17)
Not initiated ⩽ 1 month	131 (31.4)	87	44	1.39 (1.03–1.87)*

* *p* < 0.05.

CI, confidence interval; RR, relative risk.

**Table 4.** RRs for the association between the initiation phase of antipsychotic use and rehospitalization from 1 month and 3 months after discharge.

Time	*N* total (%)	*N* rehospitalized	*N* not rehospitalized	RR crude (95% CI)
**From 1 week after discharge**				
>1 week and ⩽1 month	270 (100)			
Initiated	139 (51.5)	88	51	Reference
Not initiated	131 (48.5)	87	44	1.62 (1.19–2.19)*
**From 1 month after discharge till end of follow-up**	111 (100)			
Initiated	52 (46.8)	28	24	Reference
Not initiated	59 (53.2)	39	20	1.70 (1.04–2.79)*

* *p* < 0.05.

CI, confidence interval; RR, relative risk.

Furthermore, the RR for the association between the initiation phase of AP use and rehospitalization after discharge was assessed. Patients who did not initiate their AP medication within the first week after discharge had a RR of 1.06 (95% CI: 0.82–1.38) for rehospitalization during follow up compared with those initiating during the first week. The RR for patients who did not initiate their AP medication compared with those who did initiate between the first week and first month and between the first and third month after discharge were comparable, being 1.62 (95% CI: 1.19–2.19), and 1.70 (95% CI: 1.04–2.79) respectively. Overall, patients who did not initiate their AP medication within the first year after discharge had a RR of 2.70 (95% CI: 1.97–3.68) for rehospitalization during follow up compared with those that initiated their AP medication within the first year after discharge.

During the first 3 months, 338 patients initiated their AP medication and were categorized in the different AP use categories to assess the continued drug use (implementation) and early discontinuation.

Most of the patients continued their medication with high taking compliance during the first 3 months (*N* = 225 of *N* total = 338; 66.6%). In contrast, the number of patients with low taking compliance who discontinued AP therapy increased during follow up. During the first 3 months, 225 patients continued their medication with high taking compliance; 141 of these 225 patients (62.7%) were rehospitalized at some point during the 12-month follow up (Figure 1).

Assessing the RR of psychiatric rehospitalization within the different AP use categories during the first 3 months after discharge, the results show that the RRs are all around 1 with a range of 0.98 to 1.12, and were not statistically significant (Table 5). The RR of psychiatric rehospitalization within the different AP use categories in the first year after discharge (3–12 months), remained also around 1 and not statistically significant, Table 6.

**Table 5.** RRs for the association between AP use categories (representing the continued drug use (implementation) and early discontinuation of medication use) during the first 3 months, and rehospitalization during follow up.

Time	AP use category	*N* total (%)	*N* rehospitalized	*N* not rehospitalized	RR crude (95% CI)
⩽3 months	Total	338 (100)	204	134	
	Continuers with a high taking compliance	225 (66.6)	141	84	Reference
	Continuers with low taking compliance	70 (20.7)	40	30	1.12 (0.83–1.69)
	Discontinuers with high taking compliance	28 (8.3)	13	15	0.99 (0.56–1.75)
	Discontinuers with low taking compliance	15 (4.4)	10	5	0.98 (0.52–1.87)

AP, antipsychotic; CI, confidence interval; RR, relative risk.

**Table 6.** RRs for the association between AP use categories (representing the continued drug use (implementation) and early discontinuation of medication use) within different time period and rehospitalization during follow up.

Time	AP use category	*N* total (%)	*N* rehospitalized	*N* not rehospitalized	RR crude (95% CI)
>3rd month to ⩽6th	Total	294 (100)	160	134	
	Continuers with a high taking compliance	164 (55.8)	91	73	Reference
	Continuers with low taking compliance	90 (30.6)	49	41	1.08 (0.76–1.54)
	Discontinuers with high taking compliance	18 (6.1)	10	8	0.95 (0.49–1.84)
	Discontinuers with low taking compliance	22 (7.5)	10	12	1.10 (0.57–2.13)
>6th month to ⩽9th	Total	255 (100)	121	134	
	Continuers with a high taking compliance	132 (51.8)	63	69	Reference
	Continuers with low taking compliance	76 (29.8)	36	40	1.10 (0.73–1.67)
	Discontinuers with high taking compliance	16 (6.3)	10	6	1.02 (0.52–2.00)
	Discontinuers with low taking compliance	31 (12.2)	12	19	0.87 (0.46–1.64)
>9th month to ⩽12th	Total	228 (100)	94	134	
	Continuers with a high taking compliance	114 (50.0)	47	67	Reference
	Continuers with low taking compliance	69 (30.3)	26	43	0.95 (0.58–1.53)
	Discontinuers with high taking compliance	11 (4.8)	7	4	0.94 (0.42–2.09)
	Discontinuers with low taking compliance	34 (14.9)	14	20	1.11 (0.60–2.05)

AP, antipsychotic; CI, confidence interval; RR, relative risk.

## Discussion

This study aimed to assess the association between different phases of non-adherence to AP medication and rehospitalization in patients with psychotic disorders after hospital discharge. Non-adherence was assessed for the three phases of medication use: initiation, continued drug use (implementation), and discontinuation. The results showed that not initiating AP medication compared with initiating between the first week and first month, and between the first and third month after discharge was associated with a statistically significant higher risk of rehospitalization. Furthermore, when assessing the risk within the first month after discharge, patients that did not initiate their AP medication within the first month after discharge had an almost 1.4 times higher risk of rehospitalization when compared with patients that initiated their AP medication during the first week after discharge.

The majority of the patients (63.1%) included in our study were rehospitalized during the study period, which is comparable with earlier work.^2,9,12^ Most patients, 69.3%, initiated their AP medication within 1 month after discharge. This finding is consistent with the results of Tiihonen *et al.*,^9^ who showed that 1507 of 2588 patients (58.2%) initiated their AP medication, meaning patients filled their prescription for AP medication during the first 30 days after hospital discharge.

The results of this study showed that patients who did not initiate their AP medication between the first week and first month, and between the first and third month, after discharge had a higher risk of rehospitalization. This finding is in line with earlier findings, although other studies used different adherence measures. For instance, Boden *et al.* performed a population-based cohort study that aimed to identify risk factors for rehospitalization in patients with recent onset schizophrenia or schizoaffective disorder.^25^ They reported that patients with early non-adherence, defined as not having filled a prescription of AP medication within the first week after discharge, had a higher risk of early rehospitalization than patients who were given AP medication (RR: 1.75, 95% CI: 1.13–2.72).^25^

The results of non-adherence for the continued drug use (implementation) and early discontinuations of medication use showed no trend and the results were not statistically significant in this study. Our hypothesis was that patients with low taking compliance (=MPR < 90%) who discontinued their AP medication early during follow up, had the highest risk for rehospitalization. In addition, patients with low taking compliance (=MPR < 90%) who continued their AP medication, and patients with high taking (=MPR > 90%) compliance who discontinued their AP medication early during follow up, were also expected to have a higher risk of rehospitalization compared with patients with high taking (=MPR > 90%) compliance who continued. The results of this study do not support this hypothesis. Some of the patients used long-acting injectable APs, which may have been chosen because of patients’ non-adherence. This may explain the study results. Nonetheless, previous studies did show that non-adherence to AP medication measured as gaps in prescription fills is associated with a higher risk of rehospitalization. For instance, results of two American studies, performed by Weiden *et al.* and Law *et al.*, showed a statistically significant increased risk of rehospitalization as early as the first 10 days following a missed medication refill among patients with schizophrenia.^12,21^ Additionally, a Japanese database research performed by Kuwabara *et al.*^3^ showed that patients with low taking compliance (MPR < 0.8) had almost five times (hazard ratio: 4.7, *p* value: 0.0004) higher risk of being rehospitalized. The results of this study support the notion that high taking compliance with AP medication reduces the risk of rehospitalization in patients with schizophrenia.

Non-adherence to AP medication is often not actively monitored and can be overlooked by the patients’ healthcare professionals.^4^ Community pharmacists have a unique insight into patients’ use of medication and could play an important role in intervening and signaling when patients engage in non-initiation, insufficient continued drug use (implementation), or early discontinuation.

Our results showed that patients who did not initiate their AP medication between the first week and first month, and between the first and third month after discharge had a statistically significant higher relative risk of rehospitalization. Therefore, interventions should be aimed at promoting patients to initiate their AP medication within the first month after discharge to minimize the risk for psychiatric rehospitalization. However, it remains unclear if this association between not initiating AP medication and rehospitalization was causal. Patients with more severe schizophrenia might also have a higher chance of not initiating their AP medication. Therefore, not initiating AP medication can be a symptom of the psychotic disease rather than the cause of rehospitalization.^5^

Early identification of patients with a high risk for rehospitalization is important as it allows for intensifying monitoring, which could prevent rehospitalization.^1^ Studies by our group have shown that the risk of rehospitalization can be predicted by a combination of variables from the patient/disease and medication characteristics, patients’ attitude towards medicine use, and healthcare-professional-rated assessment. These variables can be assessed relatively easily at discharge to predict rehospitalization within 6 months.^1^ Patients at risk for non-adherence to AP medication should, therefore, be identified and strategies should be developed to improve their adherence by ensuring that these patients initiate their AP between the first week and first month after discharge.

Interventions in future research could be through a more intensive cooperation between the patients’ healthcare team, as also demonstrated by other researchers.^26,27^ After a patient is discharged from the hospital, the healthcare provider at the hospital should send a prescription to the community pharmacy. The pharmacist could then disclose information to the healthcare provider about whether or not patients initiated their AP therapy (e.g., refill history). In turn, the healthcare providers can use this information to check on patients who did not initiate their AP medication to promote initiation of the AP medication between the first week and first month after discharge. For example, the healthcare provider could schedule a follow-up outpatient appointment with the patient, which commonly improves medication adherence within 5 days before and after an appointment (a phenomenon known as ‘white-coat adherence’).^6^ Lee *et al.* analyzed the association between outpatient appointment and rehospitalization in patients with schizophrenia by performing a follow-up observational study.^7^ Their results confirmed that more outpatient appointments within 60 days after discharge could lower the risk of rehospitalization within 1 year.^7^ These results, and the results of this study suggest that an outpatient appointment, to also promote initiation of AP medication, might be a promising intervention to prevent rehospitalization. Nonetheless, there is a low rate of outpatient visit. Lee *et al.* reported that only 62.6% of the patients attended their outpatient visits within 60 days after discharge.^7^ More research should be performed to investigate this hypothesis. Moreover, research on interventions and solutions to combat the low rate of actually attending outpatient follow-up appointments should be performed.

Another point of intervention could be through follow-up care after discharge performed by the community pharmacist. After discharge, community pharmacies could be informed by the psychiatric hospital when patients are discharged and expected to refill their APs. Informing the community pharmacist could be by a phone call or digital or sending a prescription for the AP medication. Either way, clear agreements about how to inform one another should be made. Interventions should consist of contacting the patient if the patient has not filled/refilled their AP medication after discharge. The community pharmacist could, for instance, contact the patient a week after discharge if the patient has not filled their prescription. The community pharmacist can provide consultation and schedule follow-up appointments as needed. At this appointment the patient can refill their AP medication and the pharmacist can give instructions and counseling about the medication and the importance of adherence.

Such interventions should be studied to see whether they prevent rehospitalization in post-discharge patients with psychotic disorder.

Further research on adherence to AP medication for initiation phase should be performed to find tailored interventions that increase adherence, and subsequently reduce rehospitalization. Interventions should be designed and evaluated through scientific research. Researchers could also focus on performing sub-analyses of, for instance, the different AP medications (e.g., side effect profile, mono- and polypharmacy, etc.).

To the best of our knowledge, this is the first study that investigated the association between adherence to AP medications by looking specifically at the different phases of medication use [i.e., initiation, continued drug use (implementation), and early discontinuation] and rehospitalization in patients with psychotic disorders. Previous studies often applied a more general classification of adherence and non-adherence to AP medication use. They did not distinguish between the different phases of AP use. Our methodology allowed for the identification of specific intervention points that can be tackled in future research to prevent rehospitalization. Another strength of this study is that stockpiling (AP refill before hospitalization) was taken into account when accessing the initiating phase, the first prescription fill after discharge.

First, when performing this observational retrospective study, there was limited data available. There was no information available on disease status, psychosocial stress, environmental factors, substance use, and reasons for rehospitalization. Thus, this study assumed that patients with a psychotic disorder should be prescribed AP medication after discharge based on the applicable guidelines, which state that patients need to continue their medication for at least 1 year after reaching remission.^8,10,22^ Since 2010, healthcare professionals have paid more attention to when patients are discharged. This may help exchange of information between primary and secondary care. However, recent research has shown that information is incompletely transferred between healthcare professionals.^28^

This study investigated adherence in the different phases according to the taxonomy of Vrijens *et al.*^23,24^ However, patients may not actually consume the medication they filled/refilled at the pharmacy. This could result in an overestimation of the MPR and an underestimation of the relative risk.

Another limitation that should be taken into account is the theoretical end date of the AP dispensing. This was adjusted if the subsequent AP medication was dispensed prior to the theoretical end date of a previous AP dispensing.^17^ However, it was unknown if the healthcare provider had increased or decreased the AP dosage. In this case, patients should have refilled their medication sooner than the adjusted theoretical end date. This could result in incorrect classification of these patients into the different time periods of initiation.

The fourth limitation of this study is that there was no information about hospitalization in other hospitals, which would have led to an underestimation of rehospitalization. Patients could have been admitted to another psychiatric hospital if they migrated to another province or when the four included psychiatric hospitals of Altrecht were full. However, this seems unlikely for the majority of the patients.

This study was performed in one region of The Netherlands. The patients’ characteristics were comparable with those of patients in other studies and other regions. In our study, olanzapine and risperidone were the most prescribed AP medications, which was also the case in previous studies from Sweden and Finland.^9,16^

## Conclusion

Patients who did not initiate their AP medication, compared with those who did in the first week and/or the first month after discharge, were associated with a statistically significant higher risk of rehospitalization. Adherence during the continued drug use (implementation) and early discontinuations of AP medication use was not associated with a statistically significant risk of rehospitalization. Therefore, interventions should be aimed at promoting patients to initiate their AP medication within the first month after discharge to minimize the chances of psychiatric rehospitalization. Additionally, tailored interventions could be explored in future studies through a more intensive cooperation between psychiatrists and pharmacists.
